# “Finding my voice again” - women’s experiences of psychological therapy in perinatal secondary care settings: a qualitative study

**DOI:** 10.3389/fpsyt.2024.1240855

**Published:** 2024-05-28

**Authors:** Jayne O’Brien, Lynsey Gregg, Anja Wittkowski

**Affiliations:** ^1^ School of Health Sciences, The University of Manchester, Manchester, United Kingdom; ^2^ Perinatal Mental Health and Parenting Research Unit, Greater Manchester Mental Health National Health Service Foundation Trust, Manchester, United Kingdom; ^3^ Manchester Health Alliance Science Centre, The University of Manchester, Manchester, United Kingdom

**Keywords:** maternal, mother-infant, psychology, change, qualitative, postpartum, peripartum, antenatal

## Abstract

**Introduction:**

Although women often experience mental health comorbidities in the perinatal period, the evidence-base for psychological therapy across diagnostic boundaries in the perinatal period remains limited. As there is a need to understand experiences of therapy, irrespective of diagnosis, to inform intervention provision, the aims of this study were to explore women’s experiences of psychological therapy for perinatal mental health difficulties and to identify the mechanisms that women attributed to the most significant therapeutic change for themselves and/or the mother-infant relationship.

**Method:**

Semi-structured interviews were conducted with 16 women who received therapy within specialist perinatal community mental health settings in the Northwest of England, the UK. Interview data were analysed using reflexive thematic analysis.

**Results:**

One overarching theme entitled participant life stories were at the heart of therapy was identified alongside three other main themes: 1.) We’re in this together – therapeutic bond and establishing a coherent sense of self, 2.) Surfing the urge to ‘fix’ feelings – Sitting with emotions improved regulation and 3.) Seeing myself in a new light – Shifting self-blame to self-compassion enhanced self-efficacy. Theme 1 consisted of three subthemes. Participants described the quality of the therapeutic relationship as the fundamental foundation to (re)connecting with their needs, values and boundaries, which improved their sense of agency, self-esteem, therapeutic engagement and self-understanding. Shifting emotional avoidance to emotional engagement improved their self-regulation. Considering alternative factors that could have contributed to their experiences helped them to defuse self-blame and enhance self-compassion. Finally, changes in their mental health led to positive relational changes in their relationship with their infant and improved communication with partners.

**Discussion:**

Sensitivity, engagement and responsivity experienced in the therapist-woman relationship was reported to be mirrored in the mother-infant relationship. Developing a coherent sense of self and self-regulation skills both appeared to heighten women’s self-compassion and empathy for their infants, which also seemed to improve their ability to tolerate uncertainty and mixed emotions within themselves and their infants. The mechanisms of change in the perinatal period are important to consider at a stakeholder, therapist and service management level to parsimoniously and best meet the needs of women and the mother-infant relationship.

## 1 Introduction

Motherhood is a time of significant psychological change which includes the development of the mother-infant relationship (1, 2). The perinatal period (during pregnancy and up to two years following childbirth) can be a joyous as well as a challenging time for women. An estimated 10-20% of women can be affected by perinatal mental health difficulties (PMHDs) (3).

Societal norms of a ‘good mother’ and societal silencing of negative experiences of motherhood can be a barrier to women making meaning of these challenging experiences (4) and seeking help for PMHDs (5, 6). The ‘good mother’ ideal can overshadow women’s distress and suffering (7) and reinforce the idea that mothers should prioritise their infant’s needs (8). In response, a mother can assume a ‘facilitator’ role in which she expects to adapt to her infant or the ‘regulator’ role in which she expects the infant to adapt to her (9). Mothers whose maternal or mothering orientation sits between these two roles and who try to find a balance between her own and infant’s needs (in the ‘reciprocator’ role) may experience a smoother transition to motherhood (10). Furthermore, external stressors (such as lack of social support, 11) and internal stressors (such as changes in identity, 5) associated with caring for a new baby can elicit feelings of inadequacy and threaten a mother’s sense of self as a woman (12, 13). Choi et al. (12, p.176) found that women strove to be “super-mum, super-wife, super-everything” in an attempt to manage felt inadequacies, which can result in greater stress in the perinatal period (14).

Poorly managed PMHDs can lead to serious consequences for the mother and infant (15). PMHDs heighten the risk of attachment difficulties between mother and infant, maternal suicidality (15–18), delayed cognitive, social and emotional development in the infant and behavioural difficulties across the infant’s lifespan (19–23). Thus, timely and effective psychiatric and/or psychological intervention for women experiencing PMHDs is essential.

Not intervening with PMHDs incurs approximately £8.1billion per annum of wider cost implications for UK society (24). Almost 75% of the costs associated with PMHDs relate to the impact that maternal mental health has on the infant’s psychosocial and psychological development (25, 26). Effective interventions in the perinatal period could improve the mother’s mental health as well as the bond between mother and infant, thereby reducing the risk of emotional difficulties in the child (27).

In 2016, the Mental Health Task Force identified that 85% of localities in England provided no or ineffective community perinatal care for women with severe PMHDs, which corroborated the need to improve access to assessment and treatment of PMHDs as a public health priority (28, 29). In response, specialist community perinatal mental health services have expanded across England (30) and, since 2018, there has been a 46% increase (from 45% to 91%) of perinatal community mental health teams (PCMHTs) meeting the recommended threshold for psychology provision of one full time clinical psychologist employed per 10,000 births (31). In 2021, 82% of PCMHTs across the UK were providing evidence-based psychological therapy (32).

The central task of clinical psychologists within PCMHTs is to collaboratively create meaning out of perinatal distress by integrating diverse psychological theories and offering high quality evidence-based psychological therapies (33). Clinical psychologists commonly offer and/or adapt interventions within a family-focused context based on a formulation to meet the highly specialised needs of women in the perinatal period (34, 35), as recommended by the National Institute of Clinical Excellence (36). Additional psychological interventions can be offered to address any problematic relationship patterns with her infant and family (33).

The evidence-base for perinatal psychological therapies draws on studies for specific PMHDs, such as anxiety, insomnia, trauma or depression (37–39). Similarly, interest in exploring women’s experiences of psychological therapy during the perinatal period has grown but it remains disorder-specific (40, 41), despite mothers often experiencing multiple comorbidities (42, 43).

Given the validity issues of diagnostic categories (44), a disorder-specific focus can limit research and lead to conceptual difficulties and possible difficulties within the therapeutic relationship (45, 46). For this reason, understanding the mechanisms by which therapeutic interventions achieve a meaningful change (47) may be more relevant for clinicians and services offering psychological support across diagnostic boundaries in the perinatal period.

Mechanisms of change are defined as “the theory driven reason that change occurs in therapy, or the how and why of the therapeutic change” (48, p.284). They are the “toothed cog” (49) p.43) that interacts between therapy techniques (e.g., challenging unhelpful thoughts), important client-therapist processes (e.g., therapeutic alliance) and the outcome/mechanism (e.g., the client engaging in more balanced thinking styles) (49, 50).

Changes in views of self through the acquisition of coping skills have been reported to be an important mechanism of change in perinatal anxiety (51) and postnatal depression (41). Hadfield et al. (52) interviewed women who reported that thought diaries, facilitated by an open and collaborative client-therapist dialogue, reduced their negative ideas of self and feelings of postnatal depression. An enhanced understanding of the mechanisms and processes that facilitate change in therapy helps clinicians, healthcare professionals and researchers to develop further and/or refine the psychological interventions offered to women, thereby improving outcomes for them, their infants and their families.

To date, reviews on the mechanisms of change in relation to perinatal psychological therapies have either exclusively focused on PMHDs in pregnancy or on anxiety comorbid with depression. In their integrative literature review of four studies, Lavender, Ebert and Jones (53) noted that mindfulness and/or mindfulness-based cognitive therapy techniques commonly led to a shift in focus towards more positive and adaptive thought patterns, increased levels of emotional and social support and improved emotional regulation skills. However, none of those four studies investigated the mechanisms of change across a range of PMHDs, nor did they highlight how mechanisms affected the mother-infant relationship. A recent systematic review of guidelines for perinatal mental health highlighted therapeutic recommendations for mother-infant dyads in perinatal mental health services were based on research that is only just emerging (54). Furthermore, Alderdice (55) indicated the need for psychological interventions to treat PMHDs and to provide help with associated caregiving difficulties, but research evaluating the impact of psychological therapies on these outcomes were limited.

The aims of the current study were to explore women’s experiences of psychological therapy and to understand the mechanisms of change in relation to their own mental health as well as their relationships with their infant and others. This understanding could then inform therapy provision, service development and potentially clinical practice guidelines for perinatal mental health. Thus, the research question we aimed to answer was: “What were women’s experiences of receiving psychological therapy for PMHDs in a PCMHT and which therapeutic processes or techniques helped or hindered them to achieve the most significant change in themselves or in the mother-infant relationship?”

## 2 Method

### 2.1 Design and ethical approvals

As part of this qualitative study, women’s experiences were explored through in-depth interviews, with interview data analysed using reflexive thematic analysis (56). This method of analysis was chosen because its theoretically flexible approach offered rich and detailed insights into the experiences and perspectives of service-users in healthcare (57).

Relevant UK National Health Service (NHS), Health Research Authority (HRA), and NHS trust approvals were obtained (NHS REC ID: 20/NW/0244). Participants provided written informed consent to participate in this study.

### 2.2 Participant eligibility

Women were included if they were aged ≥18 years, proficient in English, and had received at least four psychological therapy sessions in a PCMHT within the last twelve months. Four sessions were considered to be an acceptable dose of therapy by PCMHT-based psychologists, consulted during the planning of this study.

Participants were excluded if there was a significant risk of losing permanent custody of their infant, and/or if they were still acutely unwell (e.g., actively suicidal).

### 2.3 Recruitment

Recruitment took place across five PCMHTs in the Northwest of England between December 2021 and September 2022. Therapists in those PCMHTs informed women about the study prior to therapy ending. Letters were also sent by PCMHTs to women who had recently been discharged and who had received therapy in the last twelve months. To reach a wider group of participants, the study was advertised by perinatal charities and support groups on social media platforms (e.g., Instagram, Mumsnet). Participants were recruited through convenience sampling informed by purposive sampling (i.e., type of therapy received and level of therapist’s qualification, e.g., assistant psychologist vs clinical psychologist) to capture a diverse range of therapy experiences, and achieve greater variation in the sample (58, 59).

### 2.4 Materials and interview procedure

Participants expressed their interest either by direct email to the researcher or via the PCMHT using a consent to contact form. After participants provided written or audio-recorded consent, they provided sociodemographic and family background information (e.g., relationship status, dependents, ethnicity, employment status, current and past mental health difficulties).

Semi-structured interviews were used to encourage in-depth conversation and explore sensitive issues (60). The University’s Psychology Community Liaison Group informed the development of the interview topic guide (see **Supplementary Material** for a copy). Questions were open-ended and focused on understanding hopes for therapy, whether these were met and participants’ perceptions on the meaningfulness of client-therapist processes and therapy techniques while understanding how these mechanisms influenced change in their relationship with themselves and others.

Confidentiality was discussed with all participants from the outset. After the interview, the participants were offered a debrief sheet and £10 reimbursement for their time.

### 2.5 Data analysis

All interviews were audio-recorded and transcribed verbatim either by the lead researcher (n=7) or an approved transcription service (n=9). All identifiable information was removed and anonymised transcripts were managed using NVivo (61). Reflexive thematic analysis (56, 62) was chosen because it was compatible with the research aims and could draw out patterns of meaning as well as similarities and differences across the dataset.

Analysis started with repeated reading of transcripts promoting familiarisation with the data, which encouraged awareness of patterns in the data (63). The lead researcher then line coded and identified clusters of similar meaning across codes. During later stages of analysis, the lead researcher started to identify the implicit ideas, assumptions and conceptualisations that were hypothesised as shaping how participants explicitly described the meaning of their experiences (62, 64). While keeping the research question in mind, broader levels of meaning were recursively developed from clustered codes and this generated potential themes (58).

Development of a visual thematic map was an iterative and reflexive process that began after familiarisation with the data and concluded when final themes were generated. Definitions were generated to ensure that there was a clear organising concept for each theme and that the overall analytic story was clear (65). As final themes were organised, they were labelled by capturing reflections on the accompanying analytic story and how they related to the research question without just “…presenting summaries of data domains” (56, p.593).

### 2.6 Philosophical underpinning

#### 2.6.1 Ontology

The flexibility of reflexive thematic analysis allowed the lead researcher (JOB) to use a critical realist orientation (66) to explore how participants made sense of their experiences of the mechanisms of change and how these meanings were shaped by the wider social context.

Pilgrim (67 p.2) described critical realism as understanding that “generative mechanisms exist in the world leading to emergent properties, within, and at different levels of, reality. This causal emphasis implies that social scientists should be interested in how things come into being and change”.

#### 2.6.2 Epistemology

As we aimed to understand the mechanisms of change in perinatal psychological therapy, a critical realist ontological stance was used with a contextualist epistemology that was “concerned with truth, albeit a provisional, contextual and liminal truth”, and acknowledged social influences on the meaning both researcher and participants coproduced (68 p.179).

### 2.7 Rigour

According to Braun and Clarke (68), data saturation is inconsistent with the values and assumptions of reflexive thematic analysis. During the process of data collection and analysis, the issue of adequate sample size was reviewed based on “information power” (69) and meaning generation. According to information power (69), the more information a sample holds, relevant for the actual study, the lower the number of participants that is required for analysis. Thus, this study’s target sample was 15 to 20 participants.

In the data collection and analysis process, sample specificity was considered, ensuring there was representation of different PMHDs, types of therapy and mental health professionals facilitating therapy in the sample. Furthermore, another factor which contributed to the study’s information power was the quality of the dialogue (69). The interviewer was a trainee clinical psychologist and a doctoral student with a background in working with people across the lifespan and with a diverse range of mental health difficulties, who received training in conducting qualitative interviews, which contributed to the dialogue quality in interviews.

The lead researcher (JOB) coded the first two interviews, which were reviewed by another (LG) to assist in explanation and clarification of thinking, before coding the rest of the interview data. The research team also reviewed thematic maps to explore alternative ways of making sense of the data which had not previously been considered and to reflect on the lead researcher’s assumptions which could have impacted the analysis. For example, reflections on preconceived ideas the lead researcher had about the data during the early stages of coding, instead of allowing the narrative to be generated from the data itself.

### 2.8 Positionality

#### 2.8.1 Position within the data

During the research, the lead researcher, who was a trainee clinical psychologist, articulated and reflected on how her disciplinary assumptions and frameworks, social identity, philosophical and personal positions informed and shaped the research (68). Therefore, the lead researcher’s multiple roles as a woman, a recent first-time mother and a trainee clinical psychologist were continually considered in terms of impact on the interview questioning and interpretation of the data throughout the research process. The lead researcher considered their subjectivity as a resource during interviews and analysis (70). It guided interview questioning and responses to participants’ explicit and implicit meaning conveyed within their accounts of therapy during the perinatal period.

The lead researcher was mindful that becoming a first-time mother during the course of the study facilitated the development of rapport with participants. It allowed aspects of their own experience of being a mother to resonate with those of participants, which meant participants’ stories were listened to with even greater empathy and a genuine curiosity. The lead researcher was aware of adopting an ‘in-betweener’ positionality located somewhere on the ‘insider-outsider’ continua (71). The other researchers (AW and LG) also held multiple roles as psychologists and mothers so brought additional insight to this topic of research.

#### 2.8.2 Reflexive judgement

The potential influence of the research team’s similar positionalities on research processes (e.g., data analysis) were interrogated through reflective discussions. The lead researcher had an interest in psychological therapy, attachment and the mother-infant relationship. The lead researcher was aware that, through facilitating psychological therapy within her clinical work, she could have been inclined to focus on the positive experiences or usefulness of therapy. These positionalities, particularly the lead researcher’s clinician-researcher role, held the risk of influencing the analysis of participants’ experiences with her own preconceptions, for example, by premature conceptualisation through a clinician lens instead of staying close to the data as a researcher. The lead researcher was mindful of these beliefs and how they could impact the interview process and data analysis and used research supervision to explore these influences. Braun and Clarke (68 p.13) argue that “researcher subjectivity is an essential resource for reflexive thematic analysis”, which is why the lead researcher kept a reflective journal of thoughts for reflection, interrogation and meaning-making. Reflective discussions and journal oriented the lead researcher’s focus to meet the aims of the study as a clinician-informed researcher.

## 3 Results

### 3.1 Sample characteristics

Twenty women expressed interest in the study through their the PCMHT. Seventeen women consented to participate, 16 of whom completed the study (see **Table 1** for details). One participant partially completed an interview but subsequently withdrew due to an unforeseen issue and her interview data were not included in the analysis. Recruitment concluded after 16 interviews when sufficient repetition and depth of meaning and thematic patterning was developed across the data, relevant to the research question (72).

**Table 1:** Overview of some participant characteristics.

Pseudonym	Age of infant at time of interview	Total number of children	PMHDs	Therapy facilitated by
Zainab	12 months	3	Trauma/Anxiety	Clinical Psychologist
Eve	5 months	2	Trauma/PTSD/Auditory hallucinations	Assistant Psychologist
Betty	16 months	1	Anxiety/Depression	Systemic Psychotherapist and Clinical Psychologist
Sue	20 months	2	Anxiety/Depression/Trauma	Clinical Psychologist
Ciara	14 months	1	Anxiety/Depression	Assistant Psychologist
Jesse	12 months	1	Anxiety/Depression/Trauma	Clinical Psychologist
Morgan	3 weeks	3	Anxiety/Trauma/PTSD	Clinical Psychologist
Ebony	10 months	4	Anxiety/Depression/Trauma	Clinical Psychologist
Cerys	10 months	1	Anxiety/Depression/Trauma	Clinical Psychologist
Miriam	8 months	1	Depression/Eating difficulties	Clinical Psychologist
Aliya	12 months	3	Anxiety/Depression/Trauma	Trainee Clinical Psychologist
Leona	17 months	1	OCD - Anxiety/Depression	Support Worker
Emelia	9 months	3	Anxiety/Depression/Trauma	Support Worker
Millie	3 weeks	2	Anxiety/Depression/Trauma	Clinical Psychologist
Ava	10 months	1	Anxiety/Depression	Clinical Psychologist
Eden	12 months	2	Anxiety/Depression/Trauma	Support Worker

Interviews lasted approximately 45-90 minutes and were conducted by telephone (n=1), Zoom (n=14) or in person at the participant’s home (n=1), based on participant preference which was sometimes guided by childcare issues. Despite different interview settings, no variations in the quality of the interview data were found. Jenner and Myers (73) corroborated this and found different interview modalities did not affect the quality of data. All participants were alone or had their infant present only and had privacy while the interview took place.

Participants were aged between 26 and 43 years, with a mean average of 34 years (SD=4.8) and they were predominantly white (n=13; 81.3%). The majority had obtained an undergraduate level of education or higher (n=14; 87.5%). Eleven were married or cohabiting (75.1%), with the remainder separated/divorced or single. Participants had between one and four children, and their youngest child’s age ranged from three weeks to 20 months. Seven were first-time mothers (43.8%).

All participants received one-to-one therapy, which included compassion focused therapy (CFT; n=2), cognitive behavioural therapy (CBT; n=6), trauma-focused CBT (TF-CBT; n=1), cognitive analytical therapy (CAT; n=2), exposure and response prevention (ERP; n=1), low-intensity graded exposure (n=1), and low intensity anxiety management (n=2). Two of the 16 participants received a blended combination of therapeutic modalities, including CBT and video-interactive guidance (VIG, n=1) or CBT and CFT (n=1). Therapy was delivered by mental healthcare professionals including clinical psychologists (n=7), a combination of a clinical psychologist with a systemic psychotherapist (n=1) for CBT+VIG, and a trainee clinical psychologist (n=1). Therapeutic interventions were also facilitated by assistant psychologists (n=4) and support workers (n=3), under the supervision of a clinical psychologist. For the purpose of this paper, all mental healthcare professionals delivering psychological therapy will be referred to as therapists from this point forward. The type of therapy offered depended on the therapist’s level of qualification and expertise.

Participants described their pre-therapy PMHDs as depression with eating difficulties (n=1), anxiety with depression (n=3), OCD-anxiety with depression (n=1), trauma with anxiety (n=1), anxiety, depression and trauma (n=8), anxiety, trauma with PTSD (n=1), and trauma, PTSD and auditory hallucinations (n=1). Nine of the 16 participants (56.3%) reported having no PMHDs following therapy.

### 3.2 Findings

**Figure 1:**
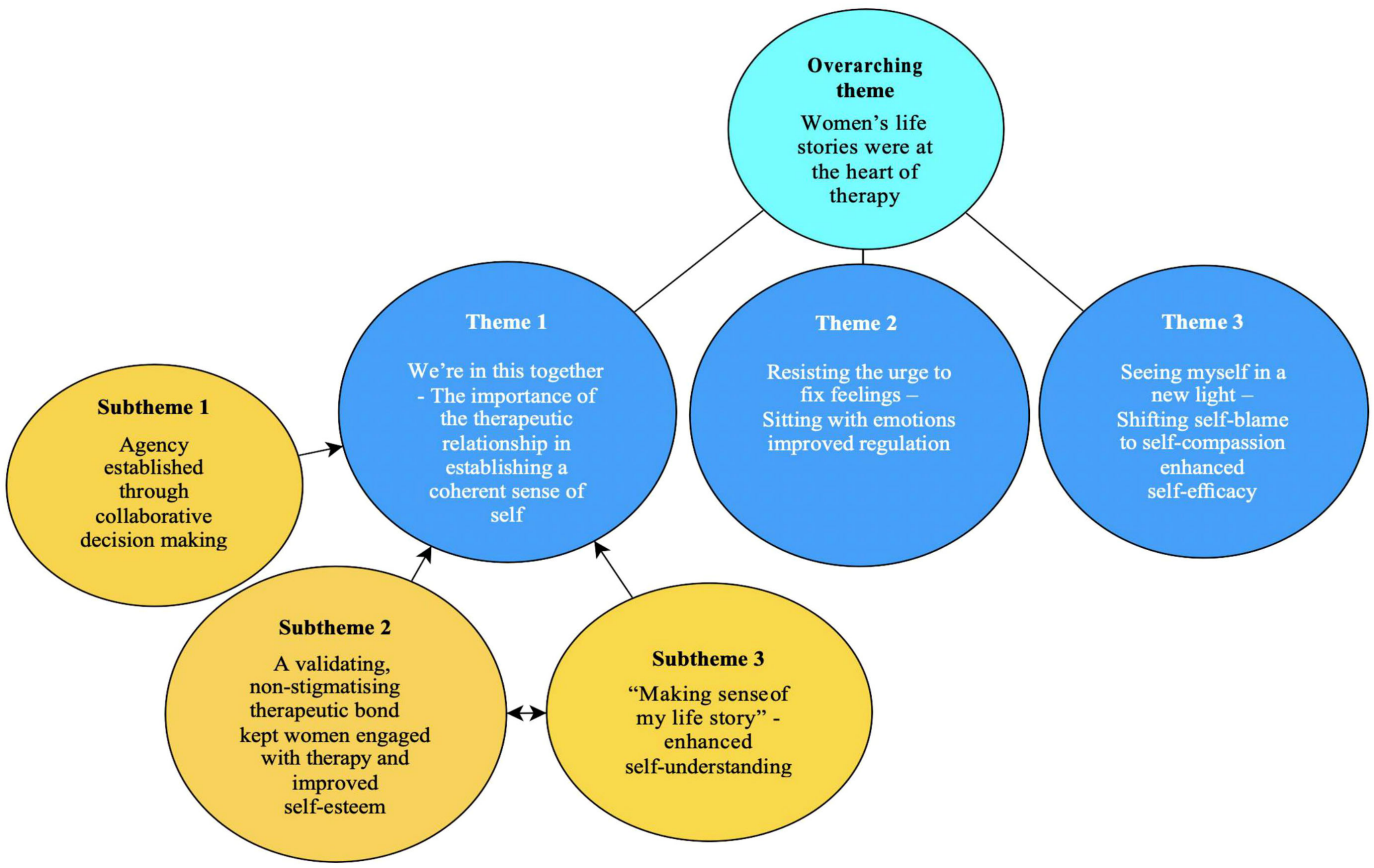
Mechanisms of change in psychological therapy for PMHDs in PCMHTs.

Analysis of the 16 interviews revealed one overarching theme *participant’s life stories were at the heart of therapy*, and three main themes: *1.) We’re in this together – therapeutic bond and establishing a coherent sense of self, 2.) Surfing the urge to ‘fix’ feelings – Sitting with emotions improved regulation* and *3.) Seeing myself in a new light – Shifting self-blame to self-compassion enhanced self-efficacy*. All participants provided data to support each theme, although perspectives were diverse. In line with the research question, **Figure 1** presents a thematic diagram of the overarching theme and three main themes and their relationship with the mechanisms of change for psychological therapy in the perinatal period.

#### 3.2.1 Overarching theme: Participants’ life stories were at the heart of therapy *^1^*

All participants talked about their perinatal experience of psychological therapy in the context of unresolved psychological trauma from their recent past or early life experiences, which can also be understood as “ghosts in the nursery” (74) p.387]. Participants valued thinking about their PMHDs through a ‘what happened to me?’ lens in therapy, which was reflected by them wanting to ‘tell their story’ in their interviews. It became evident that participants had a desire and a need to provide contextual information (e.g., birth, trauma, early life events, etc.) relevant to them when first discussing therapy. Thus, psychological therapy and its mechanisms of change in the perinatal period could only be fully understood when participants’ past experiences (e.g., childhood trauma, birth trauma, infant admitted to neonatal intensive care, pregnancy/infant loss, etc.) were considered as part of the context to their PMHDs. Most participants had limited social support.

***Theme 1: ‘We’re in this together’ – therapeutic bond and establishing a coherent sense of self***

The quality of the therapeutic relationship was fundamental to participant’s formation of a coherent sense of self. This theme explored participant’s reflections on how the therapeutic bond offered a feeling of togetherness and acted as a catalyst of change to (re)connect with their needs, values and boundaries throughout their therapy journey and in the months following discharge. Participants revealed that, by connecting with this part of their identity, it led to an improved sense of agency, self-esteem, engagement with therapy, and enhanced self-understanding – within which three subthemes were developed.

***Subtheme 1.1: Agency established through collaborative decision making***

Participants expressed that being given agency over the therapeutic process and over session content through a collaborative relationship with the therapist increased their awareness and expression of their needs in and outside the therapy room. Participants commonly expressed changes in how they overtly conveyed their needs to family and friends, particularly in terms of improved ability to share and receive support with their emotional experiences. Being given the opportunity to (re)discover their voice allowed participants to feel that they were worthy of somebody’s time and attention, much like being elevated by the therapeutic stage as Ebony described:

“She [clinical psychologist] just sort of gives me the space to just own it. And especially when I say I’m finding my voice again and talking again, she just lets me have the - it’s like giving me the stage to do all I want to” (Ebony).

Participants experienced the therapy session as protected time to reconnect with and readjust to their sense of self as a woman and as a mother. This aspect helped participants to assert boundaries when given unsolicited advice from family or friends, which led to a shift from powerlessness to a sense of agency. Participants reported that therapy helped them to feel seen and heard which reinforced the validity of their feelings, and in turn improved their mental health.

“Especially when you do feel controlled by people that care about you or are meant to care about you a lot, and then you have someone come in and just listen to you and take you seriously, is very, very empowering and very nice. [ … ] When you have a baby, especially when you’ve gone through all that, you know, you lose your identity completely. For someone to give you some time in the day to listen to you and listen to how you’re feeling rather than you listen to everybody else and looking after your baby and forgetting yourself. Like I say, very empowering” (Ciara).

The therapist being flexibly accommodating with the *“demands on the modern mum”* (Zainab) enabled participants to feel they had agency over the therapeutic process. This approach led to a common feeling that the therapeutic frame was negotiated in line with their needs and responsibilities, which helped improve their motivation to engage.

“[The [perinatal] clinical psychologist] has been really accommodating around – one day [infant] wasn’t well so I rearranged [a therapy appointment] and it was fine. The other team (community mental health team - CMHT) sort of threatened to discharge me when I had an appointment that came up that day. Just as a mum your kids are a priority, so if they’re not well or something comes up or whatever, you are going to rearrange your own appointments for them. And I just find that the Perinatal Team [ … ] were very understanding about it, whereas the CMHT were not” (Morgan).

Participants described feeling equal in the therapeutic relationship. Collaboration and choice were prioritised throughout the therapeutic process, which further consolidated their sense of agency and engagement with therapy. There was a felt togetherness in navigating the direction of therapy rather than *“force”* as Millie noted: *“I was given an option to choose what I want, like it’s not – she [clinical psychologist] did not force me to do something which I was not able to or which I was not in belief of”.*

Gaining confidence from expressing their needs in therapy helped participants to experiment with that assertiveness outside the therapy room. For instance, Aliya spoke about shifting from passivity to assertiveness to make a formal complaint about an aspect of perinatal care she received:

“I did notice while I was talking to [trainee clinical psychologist] I did things that I wouldn’t do before, so like I didn’t used to voice my opinion. I mean I always did but I stopped doing so. I didn’t complain about the service and then [trainee clinical psychologist] kind of made me want to complain about it. Not complain but I want it to change for other people, I don’t want other people to go through this, so she made me feel empowered about this stuff” (Aliya).

***Subtheme 1.2: A validating, non-stigmatising therapeutic bond kept participants engaged with therapy and improved their self-esteem***

The 16 participants reflected on how validation and normalisation of their feelings and thoughts kept them engaged with therapy. Participants expressed feeling the need to hide their inner world from others before and at the start of therapy, which commonly led to a feeling of *“overwhelm”* (Cerys) and shame, and a sense of being *“a bad mum and I shouldn’t need psychology”* (Ciara). Some participants held fears that, by sharing their PMHDs, they could be deemed unfit mothers and social services might consequently remove their infant and other children from their care.

“I still had reservations about telling the doctor ‘cos were they going to take my kids off me? Was I going to be called a bad mum? It took a lot for me to give that control over to a doctor and the perinatal team, and to trust them” (Sue).

Participants felt able to engage with therapy and *“trust the process”* (Betty) when they could relate to parts of the therapist’s character (e.g., similar humour, being a woman or a parent), which relied on the therapist being able or willing to disclose these aspects of themselves. For instance, Jesse described the authenticity of the therapeutic relationship, *“when someone else can say ‘like as a mum, that’s whatever’ - it helps, almost like it makes their understanding feel more relatable and it’s not felt like a disconnect. It’s normalised, like being a mum can be hard…”.*

Some participants shared doubts about whether they would have had the same engagement with therapy if they had a male psychologist because of the shared understanding among mothers/women of what motherhood can or could be like. However, all four participants who had a male psychologist initially shared these doubts too but were subsequently surprised by the therapeutic bond which formed.

“I think it may have helped that we were a fairly similar age and we had a similar sense of humour [ … ] I think that’s really important ‘cos I may not have opened up the same and, interestingly, he was a man. I think a lot of women would feel more comfortable talking to a woman about perinatal issues, but I was very glad that it was him because I might not have got the same outcome from somebody else” (Ciara).

Many participants described therapy as a *“safety net”* (Morgan) to explore and allow their private selves, which they had learnt to hide from others, to be seen. Participants initially felt ambivalent about sharing their PMHDs and validation from the therapist shifted this ambivalence to relief, as Eve described:

“You’re not saying these things even maybe out loud to yourself but then you’re going and sitting in a room with a stranger and you’re saying the things that are sometimes, they’re not just scary but sometimes you kind of know that actually these aren’t nice thoughts, and it’s not nice to say them out loud. [ … ] And you do feel that relief when you then get that feedback of, oh well actually somebody hasn’t recoiled at my thoughts the way I anticipated they were going to, and actually this isn’t as bad as I thought” (Eve).

The therapist’s non-judgemental character deepened participant’s sense of security to express and embrace their emotional vulnerabilities, which enabled them to explore and understand their negative self-judgements.

“[Support worker] was just so accepting and she wasn’t surprised by anything. And didn’t judge anything. And, also didn’t like ignore anything so, if you said something that you felt was an awful thing to say, she would then talk about it” (Emelia).

The data revealed that the therapist’s non-judgemental, validating responses were internalised by participants over time. Therapy provided them with a safe haven to come to when distressed, then through connection with and guidance from the therapist, participants experimented with *“re-arranging how I speak to myself”* (Sue).

“I don’t have a compassionate voice and she [clinical psychologist] was kind of teaching me how to have that compassionate voice that I was trying to emulate then after those sessions - to externalise my compassionate voice. And I wasn’t necessarily getting that from anywhere else” (Zainab).

According to participant accounts, their internalised self-validation improved their self-esteem, assertiveness with others and attunement to their infant’s and other children’s needs. Some participants described beginning to feel more emotionally present with their infant and other children.

“It helped me assert myself a bit more and play with [infant] more. Because I was scared to be alone with [infant]. It gives me a bit more, I don’t want to say courage, she is my daughter – she is not that terrifying – but that is kind of what it did. It just gave me reassurance that if she cries, it is not because you have done it and you’re a horrible person and she doesn’t want to be with you – you know, it could be this … it could be this or this” (Betty).

***Subtheme 1.3: “Making sense of my life story” - enhanced self-understanding***

Participant narratives showed that they achieved a richer understanding of themselves through formulation, which helped them to understand their PMHDs in the context of their recent and early life experiences, and *“make those connections I guess from like why … why am I thinking like this? How have I been brought up or lived that maybe these thoughts and feelings have come about”* (Ava).

According to participants, connections between early life and motherhood experiences helped them to understand the roots of their PMHDs and coping styles, thereby enhancing introspection into their relational patterns with self, infant and others.

“So he [assistant psychologist] would almost tie all of the events into one, and make me realise that, okay as a result [of childhood experiences] I’m now an adult that needs to be in control, or I’m an adult that needs to have the control, and if I don’t have the control what happens?” (Sue)

Formulations furthered participant’s understanding of how their beliefs about themselves manifested in the mother-infant relationship. For instance, *“I think one of my big triggers was rejection and I would avoid situations for fear of being rejected. Like, if I was trying to do something with [infant] and [infant] like rejected me – it was kind of like, ‘I am a bad mum’… ‘I am a bad person’”* (Betty). The insight gained from formulation helped participants to develop the skill of self-validation, which commonly reduced feelings of stigma and self-blame over time.

“I guess it’s shown me how it’s almost made me vulnerable to the trauma because I’m trying to protect [infant] from everything that I had experienced [ … ] it’s helped me to feel like, of course I felt that way because these kind of things hurt me, whereas normally I’d be like, I’m just being stupid other people have it worse. So taking into consideration that kind of background and the things that maybe made me more vulnerable to struggling after the birth, has meant a lot, it’s really helped” (Jesse).

Participants revealed that this self-understanding helped them to acknowledge and explore their *“life-story”* (Ebony), which only felt possible through the therapist holding their client’s distress using a validating, non-judgemental approach. Following discharge from therapy, written formulations continued to benefit participants by enabling them to access and reconnect with understanding the origins of their distress. Many participants spoke of how the accessibility of written formulations aided their self-regulation in the months after discharge.

“If I ever find myself stuck, I can go ‘right hang on a minute I’ll just go have a look at my [therapy] formulation and see if there’s anything that I can identify will help me right now’. [ … ] I’d feel like, wow I went through that, I dealt with it, and we did all this work and I’m here now. So if I can go through all that, I can go through whatever’s going on now. It’s really good to have, really good to have” (Ciara).

***Theme 2: ‘Surfing the urge’ to ‘fix’ feelings - Sitting with emotions improved regulation***

Many participants described lifelong patterns of emotional avoidance, a coping mechanism which worked until motherhood, when the ability to use these strategies became strained. For example, as Betty described, *“it got to the point where basically the lid just came off and they [emotions] exploded out”*. Participants outlined that therapy enabled them to navigate felt challenges of opening up about their emotional experiences and to explore *“buried”* memories.

A paradoxical feeling of relief in but unworthiness of being listened to was expressed by some, as if sharing their distress was a burden to others. Participants stated that therapists encouraged them to engage with this discomfort in an attempt to consider the value of opening up.

“It’s like I’m taking up space and other people’s time. And maybe with [clinical psychologist] she shows you that you’re not doing that and you need … there is a reason why you’re having these conversations. And helping you understand why you need to have … why you need to think about these things” (Ava).

Many participants expressed ambivalence about exploring their feelings. By collaboratively contemplating the possible consequences of emotional avoidance, participant’s motivation to engage with their emotional experiences and the therapy process increased.

“By session three I started to think, what am I even getting out of this? (…) then [assistant psychologist] asked me a question about burying my emotions and thoughts around that. And whether I felt that would be helpful for me in order to continue through life basically, which then made me think, I can’t do that because every time I think about what happened in my pregnancy, or when [infant] was born, it just filled me with emotions [ … ] and that [burying emotions] is not that helpful and that they’ll probably come up at some other point in life” (Cerys).

According to participants, therapists commonly coregulated their emotional distress by encouraging them to *“engage with the emotion”* (Jesse) and normalising that *“it’s okay to have those feelings, and that’s not a problem”* (Emelia). Normalising feelings encouraged hope and optimism about therapy outcomes and scaffolded participant’s self-regulation skills. Participant narratives demonstrated that this aspect changed their relationship with emotional experiences and improved acceptance of emotions felt.

“It’s always been a bit of a coping mechanism, but especially with the trauma - not wanting to feel an emotion, wanting to problem solve it or distract it. So, we’ve been doing work on that and kind of trying to be the person I’d be for someone else I guess. Or trying to validate it and empathise with myself and give myself a minute [ … ] it’s helped me definitely to reduce my anxiety and then in turn that’s helped me to improve my mood” (Jesse).

Self-regulation skills enabled participants to tune into and coregulate their infant and other children’s distress more effectively, which further improved their self-efficacy. For example, *“yesterday my four-year-old was just crying and screaming. Before I would have screamed at him, “Stop screaming, stop crying.” But I was so calm to say, “Come to mummy, what is it? Tell me,” you know. So I’m not on the edge like that as much”* (Ebony).

Participants also began to share their emotional experiences with friends and family, including with their infant and other children. Participants described a desire to change intergenerational patterns of how emotions were experienced and interpreted. Participants wanted to encourage their infant and other children to embrace emotional experiences and to share their emotional experiences freely. Participant accounts indicated that involving partners improved their communication and openness to talking about feelings, thoughts and needs, which further supported participant’s emotional regulation outside the therapy room.

“It has improved communication with me and my husband. We’re far more honest, the second time round, I was able to tell my husband I’m hearing things again and I’m frightened” (Eve).

***Theme 3: ‘Seeing myself in a new light’ - Shifting self-blame to self-compassion enhanced self-efficacy***

Through guided discovery, participants revealed that they considered their present and past lives through an alternative lens and, with the therapist’s *“gentle nudging”* (Ebony), they contemplated other ways of viewing the same situation both in and outside the therapy room. This cognitive process gradually enabled participants to enhance their curiosity for alternative factors that could have contributed to their emotional experiences, which helped to defuse participants’ self-blame.

“You kind of hold onto these things, and feel bad about the past, for instance, and when somebody else kind of mirrors them in a different way then it helps you understand it more and not feel bad about it. And that also has helped me perhaps think more in that way as well. Trying to look at other situations for what else might be happening that is making me feel that in that situation. So I feel like I am trying to implement her way of thinking into other areas” (Ava).

According to participants, this restructuring of their beliefs about themselves increased self-forgiveness and/or acceptance. Considering alternative perspectives also helped participants to feel more assertive, namely by placing and holding boundaries in their relationships with family and friends, which evoked an awareness of improved self-esteem and self-worth.

“So changing that mindset did help with what I’m prepared to put up with from people. So like my family are quite critical and I’m more minded to take that opportunity to reflect back on the person if they’re being harsh, you know, instead of explaining myself. So that’s always been the dynamic that I’ve had, that I always feel like I have to apologise, and it’s kind of recognising, well actually no, the thing that that person just said was really inappropriate or snarky and I don’t need to explain myself” (Zainab).

Some participants spoke about practicing skills between sessions and how it influenced their self-efficacy. Whilst navigating relational challenges was explored and scaffolded in therapy, one of the challenges some participants faced was practicing assertiveness skills outside the therapy room. Operationalised between-session goals helped participants to consolidate their relational skills.

“Having those goals set towards the end of each session - right discuss it with [name] or go away or speak to [name] about what we’ve discussed in the session today, actually might not sound very much at all but it’s actually a huge barrier. So [ … ] rather than just starting a new session this time, sort of checking back in on the last session. I guess as well to link it all together and to provide that bridge between the sessions” (Cerys).

Many participants found their internal voice shifting from a critical to a compassionate or nurturing tone, which improved self-esteem and enabled participants to prioritise their own needs and values. This emotional shift helped participants to regain a sense of self-efficacy in relationships with family, friends and their children.

“Just navigating relationships and challenges because I’ve always been very much a people pleaser and feeling I’m not good enough, so they’re right. So actually being like, no, and trying to have people listen to me and what my values are I suppose, rather than what theirs are. [ … ] It’s given me time to actually think about what I want and what I want to do rather than what someone else wants me to do” (Ciara).

Most participants described how understanding and exploring their relationship with themselves significantly enhanced their enjoyment and fulfilment of the emotional bonds with their infant, children and others. Betty described her journey from the darkness of suicidal ideation and debilitating self-blame to the lightness of enhanced self-worth and subsequent new beginnings with her family.

“Knowing that if I am getting those [suicidal] thoughts, I can see now - it sounds stupid - I couldn’t see at that time what was around me, but now I can. I look at my daughter and my husband, and feel how lucky I am to have them. How amazing they are and I have no time to think of those [suicidal] thoughts anymore, and have no reason to think of those thoughts anymore because everything I have got and everything that I wanted is right in front of me” (Betty).

## 4 Discussion

This study was the first to understand participant’s experiences of psychological therapy irrespective of diagnosis and therapeutic modalities in specialist PCMHTs. Participants’ narratives revealed one overarching and three distinct, yet interconnected themes which could only be fully understood when participant’s past or early relationships, referred to as “ghosts in the nursery” (74) p.387], were considered. Shifting self-blame, establishing a coherent sense of self (through the therapeutic bond, collaboration, and formulation) and engaging with difficult emotions were identified as potential mechanisms which led to an improved sense of agency, self-understanding, self-efficacy, and self-regulation. Participant accounts demonstrated that positive relational change was experienced for themselves and their relationships with their babies.

Unsurprisingly, some of these findings had already been identified in the wider psychological literature on therapy. For example, the therapeutic relationship, the development of self-regulation skills and enhanced self-understanding have previously been reported as key mechanisms in therapeutic change (e.g., 75–79). However, women in the perinatal period have additional needs as primary caregivers of an infant, and perinatal mental health services have equal responsibility for the infant and mother to improve long-term outcomes for the mother-infant relationship and cost-savings to society (80).

Participants’ narratives revealed that they experienced extreme inadequacy, guilt and stigma when their experiences did not match the idealised depictions of motherhood, which are known barriers to accessing perinatal mental health support (81). As Choi et al. (12, p.176) identified that women, who did not have PMHDs, strove to manage felt inadequacies by being “super-mum, super-wife, super-everything”, it could be argued that participants in this study felt additional internalised pressure to conceal their PMHDs. However, the findings showed that formulation and normalisation of their PMHDs encouraged a more realistic expectation of motherhood and the mother-infant relationship in these participants, which reduced their self-blame and instead improved their self-esteem and attunement with their infant.

A striking pattern across participants’ accounts was the importance of the therapeutic relationship as an engagement process, which could be explained by attachment theory (82). Therapy acted as a safe haven or secure base, which allowed participants to engage with therapy and explore painful or traumatic psychological experiences. This process involved the therapist coregulating their client’s distress and provided adaptive emotional experiences to reconfigure their internal working models (IWM), which has been reported in the wider psychological literature [e.g (83–86). Furthermore, the findings indicated that, like an attachment relationship (87), participants internalised the therapist’s validation and normalisation of their experiences which empowered participants to feel they were ‘good enough’ mothers. Participants’ past experiences were unchangeable and influenced their perceptions of self as a mother, but therapy facilitated the development of alternative perspectives so that they were able to control current factors contributing to their PMHDs.

This sensitivity, engagement and responsivity (or mind-mindedness) experienced in the therapist-woman relationship was then reported to be mirrored in the mother-infant relationship and improved a mother’s felt ability to connect with and soothe her infant’s emotional experiences. This maternal ability to be mind-minded with her infant in the first year of life is considered to be important for enhancing the quality of early mother-infant interaction, which can improve the security of the mother-infant bond and eventual attachment (see 88, 89). This study highlights the usefulness of attachment theory (82, 86, 90) in understanding and complementing therapeutic processes and outcomes for PMHDs while holding the mother-infant relationship in mind. Similarly, the usefulness of integrating attachment theory into therapeutic processes irrespective of therapeutic modality has been reported in the non-perinatal literature (91).

Findings on improved agency, self-understanding, validation, and emotional regulation could be further explained by self-regulation theory (e.g., 92). Therapists encouraged a collaborative approach throughout the therapeutic process which improved a participant’s sense of agency and self-esteem. Self-validation and regulation helped participants to withstand the challenges of motherhood. Sanders and Mazzuchelli (92) argue that parents can improve their self-regulation skills by appreciating their strengths and weaknesses, and achievements and setbacks, without being unhelpfully critical. Participants similarly highlighted the connection between improved self-compassion and self-regulation in this study.

Developing a coherent sense of self and self-regulation skills both heightened self-compassion and empathy for their infants, which improved their ability to tolerate uncertainty and mixed emotions within themselves and their infants. The ability to accept conflicting feelings is deemed to be protective and important for the flexible reciprocity in meeting the emotional needs of *both* mother and infant (the ‘reciprocator’ role) (10). Our findings indicated that therapy elicited a number of psychological shifts in participants from initial guardedness to openness, self-blame to self-compassion, dismissiveness to self-validation, and from the perfect to good enough mother. These changes in women’s emotional experiences could over time lead to a wider societal shift from the ‘perfect mother’ to the ‘good enough’ mother (93), which could reduce stigma associated with PMHDs.

This study offers practice-informed insights to the growing evidence-base for psychological therapies in the perinatal period. Although, the findings of this qualitative study cannot be generalised, they could be transferred to other contexts and countries. Certain aspects of psychological therapy for PMHDs appear to be particularly pertinent for the therapeutic engagement and outcomes for mothers of a young infant. These findings will be relevant for the development or updating of guidelines for perinatal psychological therapy, especially within the UK and have important implications for future research on psychological interventions offered to women in the perinatal period.

### 4.1 Clinical and policy implications

**Figure 2:**
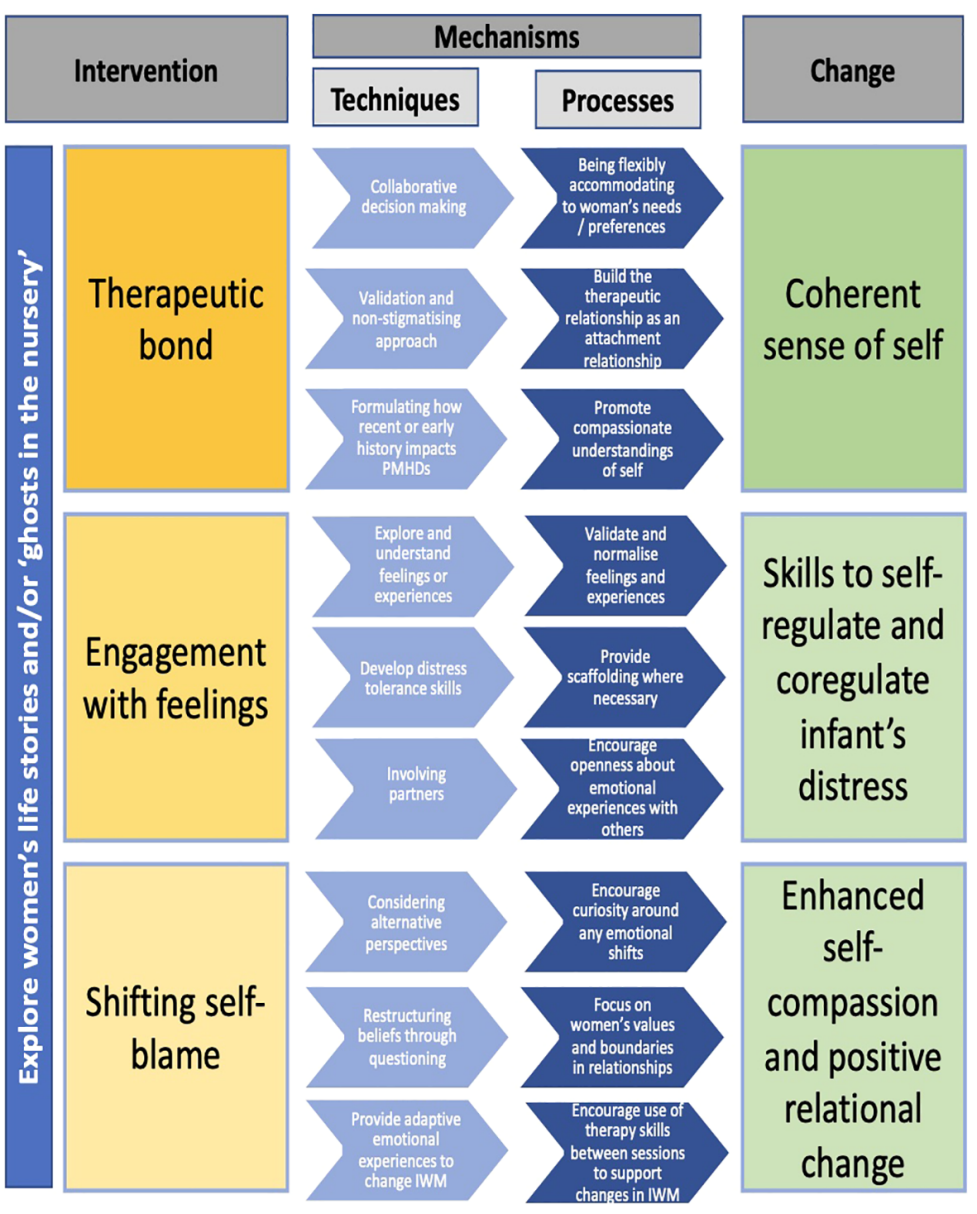
Mechanisms of change model for perinatal psychological therapy.

Participants described and almost outlined the processes in perinatal psychological therapy which elicit change in women’s mental health and the mother-infant relationship. When these are combined with established therapy techniques, clear recommendations were revealed and are outlined in **Figure 2**. This proposed model outlines the relationship between the pertinent aspects of psychological intervention in the perinatal period, the proposed mechanisms (i.e., techniques and processes), and the therapeutic change based on commonality of participant experiences. Although this model is possibly most relevant for healthcare professionals delivering psychological therapy in the perinatal period, it could be used between therapists and women as a guide for the direction of therapy or as a resource for the formulation of therapeutic goals. Kazdin (94, p.4] highlights that “understanding the mechanisms of change can bring order and parsimony to the current status of multiple interventions”. Therefore, the model provided in **Figure 2** could offer stakeholders (e.g., women, healthcare professionals, service leads, etc.) a framework to inform psychological therapy provision (irrespective of PMHDs and therapy modality) in PCMHTs. **Figure 2** could inform policies for the delivery of perinatal mental health care by broadening recommendations to focus on the mechanisms by which therapy elicited change for participants in this study.

### 4.2 Strengths and limitations and future research suggestions

To our knowledge, this is the first study to explore the mechanisms of change for psychological therapy in perinatal mental health settings. Strengths of this study include the robust procedure for data analysis. However, some limitations have to be acknowledged: women who took part in this study were predominately white, heterosexual and relatively well educated. This is a known limitation of research studies and may compromise the transferability of findings to different contexts. External, non therapy-related, factors could have influenced the psychological changes reported by participants in this study. For instance, encouragement to engage with therapy or changes in support offered by family and friends could have occurred as a parallel process to therapy. The findings were restricted to a UK/English setting and may not be transferable to other health care systems and other countries.

Future research investigating psychological change in the perinatal period should aim to address the above-mentioned limitation regarding diversity issues within the sample, including mothers who are not proficient in English. Most of the mothers received psychological therapy during the first year post-birth. However, commissioning of PCMHTs is currently changing and services will increasingly be working with mothers up to two years following birth. Therefore, it would be helpful to capture women’s experiences of psychological therapy received between 12-24 months following birth to consolidate our understanding of the mechanisms of change in the perinatal period.

### 4.3 Conclusions

This novel study explored the mechanisms of change within psychological therapy irrespective of mental health diagnosis or PMHDs and therapeutic modality. Findings demonstrate that shifting self-blame, focusing on the therapeutic relationship and formulation, and engaging with feelings were mechanisms which participants reported helped them improve their self-understanding, self-compassion and self-regulation skills while considering the mother-infant relationship. At a time when PCMHTs are rapidly expanding across England, this study offers a mechanisms of change model which could be used as a framework to improve the facilitation and outcomes of evidence-based psychological therapies in PCMHTs. 

## Data availability statement

The raw data supporting the conclusions of this article will be made available by the authors, upon reasonable request.

## Ethics statement

The studies involving humans were approved by Relevant UK National Health Service (NHS), Health Research Authority (HRA), and NHS trust approvals were obtained (NHS REC ID: 20/NW/0244). The studies were conducted in accordance with the local legislation and institutional requirements. The participants provided their written informed consent to participate in this study. Written informed consent was obtained from the individual(s) for the publication of any potentially identifiable images or data included in this article.

## Author contributions

All authors conceived of and participated in the design of the study, based on an idea by AW. JO coordinated recruitment, data collection and performed the reflexive thematic analysis. All authors reflected on the interpretation of the findings and conclusions. JOB drafted the manuscript, which was reviewed and revised by AW and LG. All authors read and approved the final manuscript.

## Footnotes

1 Theme names were chosen by the research team that best fit the central organizing concepts. They were a combination of popular culture sayings and short quotes from interviews.
