# Supplementary material for: “Finding my voice again” - women’s experiences of psychological therapy in perinatal secondary care settings: a qualitative study
Source: Front Psychiatry. 2024 May 28;15:1240855. doi: 10.3389/fpsyt.2024.1240855 (PMC11165924; doi:10.3389/fpsyt.2024.1240855)
Supplement: Supplementary file 1 [file Table_1.docx]

**TOPIC GUIDE**

| ***TOPIC*** | ***MAIN QUESTIONS*** | ***ADDITIONAL QUESTIONS*** | ***CLARIFYING QUESTIONS*** |
| --- | --- | --- | --- |
| **EXPERIENCE of psychological therapy (AS A MUM)**  **Pregnancy & early motherhood as ‘window of change’** | What kind of psychological therapy did you have? … And what was it for? | What were your hopes and expectations for therapy as a mother?  When you cast your mind back to your experience of psychological therapy, what comes to mind? What do you remember? | Tell me more about…  [that] / [insert comment from interview]  Mindful that you had therapy after becoming a mum (for x time) and wondering how it felt to have therapy at this time in your life? |
| **MEANINGFULNESS of therapeutic techniques or processes (AS A MUM)**  **Pregnancy & early motherhood as ‘window of change’**  **Keep in mind… “toothed cog” interacting between:**   - **Techniques** - Important **client-therapist techniques**   **Outcome/mechanisms** | Techniques / client therapist dynamics / outcome Q:  When you think about the techniques or skills learnt in your therapy sessions, what comes to mind? | *Client-therapist techniques:*  What parts of therapy felt particularly meaningful and what were the reasons for this?  What is it about the relationship between you and your therapist that felt important as a mother? | Tell me more about…  [that] / [insert comment from interview]  What was it about having therapy through a PCMHT that kept you engaged with it? |
| **CHANGE through therapy**  **(AS A MUM)**  **Pregnancy & early motherhood as ‘window of change’**  **Keep in mind… “toothed cog” interacting between:**   - **Techniques** - Important **client-therapist techniques** - **Outcome/mechanisms** | *Outcome/mechanism Q:*  Where did you notice changes in your life during or after therapy?  *Follow up:* How did this change happen? | *Techniques / client therapist dynamics / outcome Q:*  What was helpful about the therapy you had?  *Follow-up:* How did you know it was useful?  What parts of therapy helped you to achieve the most meaningful change in yourself or your bond with your baby?  *Follow up:* How did this change come about?  How did your therapist help you to achieve this change? | *Techniques / client therapist dynamics:*  What influence did therapy have on your mood / relationships (e.g., with baby / partner / friends / family?)  If stuck…  Some women we have spoken to say that these changes are… (e.g. creating balance in their feelings or thoughts)  and these happened by… (e.g. skills building – mindfulness, or challenging unhelpful thoughts, or being seen and heard by a non-judgemental, warm, compassionate therapist). Are these something you have experienced? |
